# Burden of Diabetes Mellitus in Nepal: An Analysis of Global Burden of Disease Study 2019

**DOI:** 10.1155/2022/4701796

**Published:** 2022-12-20

**Authors:** Achyut Raj Pandey, Krishna Kumar Aryal, Niraj Shrestha, Dikshya Sharma, Jasmine Maskey, Meghnath Dhimal

**Affiliations:** ^1^HERD International, Kathmandu, Nepal; ^2^Public Health Promotion and Development Organization, Kathmandu, Nepal; ^3^Abt Britain, Kathmandu, Nepal; ^4^Sambhav (Possible), Kathmandu, Nepal; ^5^Oxford University Clinical Research Unit, Lalitpur, Nepal; ^6^Nepal Health Research Council, Kathmandu, Nepal

## Abstract

Globally, the number of people living with diabetes mellitus (DM) increased by 62% between 1990 and 2019, affecting 463 million people in 2019, and is projected to increase further by 51% by 2045. The increasing burden of DM that requires chronic care could have a considerable cost implication in the health system, particularly in resource constraint settings like Nepal. In this context, this study attempts to present the burden of DM in terms of prevalence, mortality, and disability adjusted life years (DALYs). The study is based on the Global Burden of Disease Study 2019, a multinational collaborative research, led by the Institute for Health Metrics and Evaluations. In the study, the overall prevalence of DM was estimated using DisMod MR-2.1, a Bayesian metaregression model. DALYs were estimated summing years of life lost due to premature death and years lived with disability. There were a total of 1,412,180 prevalent cases of DM, 3,474 deaths and 189,727 DALYs, due to DM in 2019. All-age prevalence rate and the age-standardized prevalence rate of DM stood at 4,642.83 (95% uncertainty interval (UI): 4,178.58-5,137.74) and 5,735.58 (95% UI: 5,168.74-6327.73) cases per 100,000 population, respectively, in 2019. In 2019, 1.8% (95% UI: 1.54, 2.07) of total deaths were from DM, which is a more than three-fold increase from the proportion of deaths attributed in 1990 (0.43%, 95% UI: 0.36, 0.5) with most of these deaths being from DM type 2. In 2019, a total of 189,727 disability adjusted life years (DALYs) were attributable to DM of which 105,950 DALYs were among males, and the remaining 83,777 DALYs were among females. Overall, between 1990 and 2019, the DALYs, attributable to Type 1 and 2 DM combined and for Type 2 DM only, have increased gradually across both sexes. However, the DALYs per 100,000 attributable to DM have slightly reduced across both sexes in that time. There is a high burden of DM in Nepal in 2019 with a steep increase in the proportion of deaths attributable to DM in Nepal which could pose a serious challenge to the health system. Primary prevention of DM requires collaborative efforts from multiple sectors. Meanwhile, the current federal structure could be an opportunity for integrated, locally tailored public health and clinical interventions for the prevention of the disease and its consequences.

## 1. Introduction

Globally, a little under half a billion people (463 million) were estimated to have been living with diabetes mellitus (DM) in 2019 [1]. Between 2009 and 2019, the number of people living with the disease increased by 62%. Moreover, by 2030, the number is projected to increase by 25% and by 51% by 2045 [1]. Although about a tenth (9.3%) of the global adult population aged 20 to 79 years are living with the disease, the prevalence rate in low-income countries was much lower at 4% [1]. In contrast, its prevalence in Nepal was estimated at 8.5% in 2019, with a higher prevalence in the population over 60 years (13.3%) and among males (11%) [2].

DM is a metabolic disease that affects how the body converts food into energy and is marked by elevated glucose levels in the blood (or blood sugar) [3, 4]. It may be the result of the body being unable to produce any or enough insulin that the body requires or because the insulin produced by the body could not be used effectively [3]. There are two subtypes of DM: DM type 1 (previously known as insulin-dependent diabetes) and DM type 2 (previously known as noninsulin-dependent or adult-onset diabetes mellitus).

Among people with DM in Nepal, only 52.7% were aware of their DM status [5]. This is particularly concerning given the consequences. DM can cause severe damage to various parts of the body, including the heart, blood vessels, kidneys, eyes, and nerves [4]. Long standing and poorly controlled DM can pose an increased risk of cardiovascular illness, renal disorders, neurological diseases, and cognitive and psychiatric illness. Visual problems like retinopathy, cataract, and glaucoma have also been linked to DM [4]. Diabetic retinopathy is responsible for 2.1% of global blindness [6].

DM poses a considerable cost implication on the health system throughout the world. Approximately US$ 760 billion, which constitutes about 10% of total health expenditure, was spent on the care of DM in 2019 [7]. Globally, the DM-related expenditure is projected to increase and will reach US$ 825 billion by 2030 and US$ 845 billion by 2045 [7]. Apart from the direct cost, DM can also have indirect costs associated with loss of labor force productivity and absenteeism [7]. The mean per capita spending for DM ranged from US$ 20.9 to US$ 545.2 in low-income countries and low- and middle-income countries with a median per capita expenditure of US$ 116.4 [8].

According to the Centers for Disease Control and Prevention, the odds of depression among people living with DM are two to three times higher than those without it [9]. Chronic diseases such as DM are demanding and take a “psychological toll” on people living with it [10]. For instance, the term “diabetes distress” has been used to refer to the physical and emotional burden associated with self-management of the diseases such as the continuous requirement to monitor and receive treatment including concerns about complications [11, 12]. Further, fear of a hypoglycemic experience of serious nature can be traumatic for the individual and family alike [10]. Psychological distress among people living with DM has been reported following the recommendation by health care providers to add insulin to the DM regimen. [10] For people living with DM, this may indicate a failure to manage their condition using noninsulin antihyperglycemic drugs, and the fear arising from self-injection of insulin is another cause of distress [13, 14].

The increasing burden of DM could pose a serious challenge to the health system in developing countries due to complications resulting from the disease, cost of disease treatment, lack of human and financial resources, and lack of awareness about the disease at the population level and among patients [15]. The increasing burden could also partly offset the achievement in reducing overall mortality in the country resulting from a decline in mortality rate, particularly those resulting from the decline in communicable, maternal, neonatal, and nutritional diseases or could at least slow down the pace of decline in mortality. Therefore, countries need to have a clear understanding of the burden of DM to design effective strategies to reduce the occurrence and consequence of the disease, thereby reducing the burden and mortality resulting from such diseases. In this context, this article examines the burden of DM, disaggregated by sex from 1990 to 2019 in Nepal. Although some of the previous studies have provided data on the prevalence of DM in the Nepalese context [2, 16], a comprehensive picture of the burden of the disease in terms of deaths caused by the disease and the disability-adjusted life years (DALYs) due to DM is not available. This study attempts to provide a comprehensive picture of the burden of DM in Nepal.

## 2. Materials and Methods

This analysis is produced based on the estimates from the Global Burden of Disease Study (GBD) 2019 using the data publicly available in the “GBD Compare” data visualization portal operated by Institute for Health Metrics and Evaluations (IHME) [17]. GBD 2019 is a multinational collaborative research study led by IHME that estimated the burden of disease for 204 countries and territories from 1990 to 2019 [18]. The GBD study uses a total of 86,249 data sources globally, including sources like censuses, household surveys, health service utilization data, civil registration and vital statistics, disease registries, air pollution monitors, satellite imaging, disease notifications, and other sources from 1990 to 2019 in making estimates of the disease burden. A total of 389 data sources were used from Nepal in GBD 2019.

In the study, DM was defined as having a fasting plasma glucose ≥ 126 mg/dL (7 mmol/L) or reported to be on treatment with drugs or insulin for DM, or persons < 15 years of age diagnosed by physicians and identified through hospital records or a diabetic registry. The overall prevalence of DM was estimated using DisMod MR-2.1, a Bayesian metaregression model. DisMod-MR produces estimates of the prevalence of DM by age, sex, and year [19].

GBD researchers estimate adult and child mortality using data from censuses, vital registration systems, and periodic and household surveys. Years of life lost due to premature death (YLLs) from different diseases are calculated based on data from vital registration with medical certification (or cause of death assessed by verbal autopsies where medical certification of the causes of death is not available). Years lived with disability (YLDs) are calculated using outpatient and inpatient data from facilities, cancer registries, and direct physical measurements and examinations. Once YLLs and YLDs are estimated, disability-adjusted life years (DALYs) are estimated, summing up the YLLs and YLDs [20].

In GBD 2019, 369 diseases and injuries were organized into a levelled cause hierarchy from the three broadest causes of death and disability at level 1 to the most specific causes at level 4 [19, 21]. Level 1 causes include aggregates of noncommunicable diseases (NCDs); injuries; and a broad category combining communicable maternal, neonatal, and nutritional diseases (CMNN diseases). At level 2, there are 22 disease and injury aggregate groupings such as cardiovascular diseases, respiratory infections, tuberculosis (TB), and transport injuries. Level 3 includes specific causes such as stroke, hypertensive and rheumatic heart disease, and peripheral artery disease.

For this article, we extracted data from level 3 for the trend in prevalence, mortality, and DALYs attributable to DM. Data for DM type 1 and DM type 2 were obtained from level 4. We have reported both all-age and age-standardized rates in this article. Age standardization is a statistical technique that makes the rates comparable across the populations with different age structures. In the study, age standardized populations were calculated using GBD world population age standard. Nonweighted means were used for generating a standard population age structure for all countries and territories. Details of the age-standardization process have been elaborated in one of the previous publications from GBD study [19].

## 3. Results

A total of 1,412,180 prevalent cases (744,212 cases among males and 667,968 cases among females) of DM, 3,474 deaths (1,796 deaths among males and 1,678 deaths among females) and 189,727 DALYs (105,950 DALYs among males and 83,777 DALYs among females) were due to DM in 2019 (not in table).

### 3.1. Prevalence of DM

All-age prevalence of DM increased from 1,861.58 (95% uncertainty interval (UI) F: 1,678.6-2,058.88) cases per 100,000 population in 1990 to 4,642.83 (95% UI: 4,178.58-5,137.74) cases per 100,000 population in 2019.

In the same period, the age-standardized prevalence rate of DM increased from 3,192.87 (95% UI: 2,883.68-3,516.35) cases per 100,000 population to 5,735.58 (95% UI: 5,168.74-6,327.73) cases per 100,000 population, an approximate 80% increase in the rate. Among the two types of DM, DM type 2 was more common with all-age prevalence rate of 4,440.84 (95% UI: 3,983.96-4,937.08) cases per 100,000 population in 2019, which was increased from 1,707.06 (95% UI: 1,520.34 1,918.68) cases per 100,000 population in 1990. In the same period, the age-standardized prevalence rate of DM type 2 increased from 3,005.07 (95% UI: 2,697.35-3,332.37) cases per 100,000 population to 5,524.09 (95% UI: 4,969.32-6,119.99) cases per 100,000 population. Similarly, all-age prevalence of DM type 1 increased from 154.53 (95% UI: 118.22-197.56) cases per 100,000 population in 1990 to 201.99 (95% UI: 156.94-255.48) cases per 100,000 population in 2019. The age-standardized prevalence rate of DM type 1 increased from 187.79 (95% UI: 146.09-237.53) to 211.49 (95% UI: 165.51-265.12) cases per 100,000 population. In 2019, all-age prevalence stood at 5,133.27 (95% UI: 4,609-5,694.48) cases per 100,000 population in males, while age-standardized prevalence was 6,440.78 (95% UI: 5,762.38-7,144.47) prevalent case per 100,000 population. Similarly, all-age prevalence and age-standardized prevalence rate among females were 4,196.16 (95% UI: 3,734.8-4,678.26) cases per 100,000 population and 5,125.94 (95% UI: 4,573.8-5,707.68) prevalent cases per 100,000 population (Table 1).

### 3.2. DM Mortality

The all-age mortality rate for DM has more than doubled between 1990 to 2019 and increased from 4.85 (95% UI: 3.93-5.99) deaths per 100,000 population in 1990 to 10.06 (95% UI: 8.29-12.11) deaths per 100,000 population in 2019. The age-standardized mortality rate increased from 12.05 (95% UI: 9.63-14.75) deaths per 100,000 population, and 19.57 (95% UI: 15.5-23.58) deaths per 100,000 population. The all-age mortality rate for DM type 2 increased from 4.11 (95% UI: 3.27-5.14) deaths per 100,000 population to 10.67 (95% UI: 8.43-12.99) deaths per 100,000 population and the age-standardized mortality rate increased from 11.09 (95% UI: 8.83-13.74) to 18.66 (95% UI: 14.66-22.57). Both the all-age mortality rate and the age-standardized mortality rate for DM type 1 have remained stable in the period. Both the all-age mortality rate and age-standardized mortality rates for both types of DM are similar among males and females.

In 2019, 1.8% (95% UI: 1.54-2.07) of deaths were from DM, which is more than three folds increase from the proportion of deaths attributed in 1990 (0.43%, 95% UI: 0.36-0.5). The proportion of deaths attributable to DM type 2 increased from 0.36 (95% UI: 0.3-0.44) in 1990 to 1.68 (95% UI: 1.44-1.95) in 2019 while that for DM type 1 increased from 0.07 (95% UI: 0.04-0.09) in 1990 to 0.12 (95% UI: 0.09-0.16) in 2019. In males, 1.7% (95% UI: 1.35-2.05) of deaths in 2019 were due to DM, which is an increase from 0.48 (95% UI: 0.38 - 0.58) in 1990. Similarly, in females, 1.91% (95% UI: 1.56-2.34) of deaths in 2019 were due to DM, which increased from 0.37 (95% UI: 0.28-0.48) in 1990 (Table 2).

### 3.3. DALYs Related to DM

**Figure 1:**
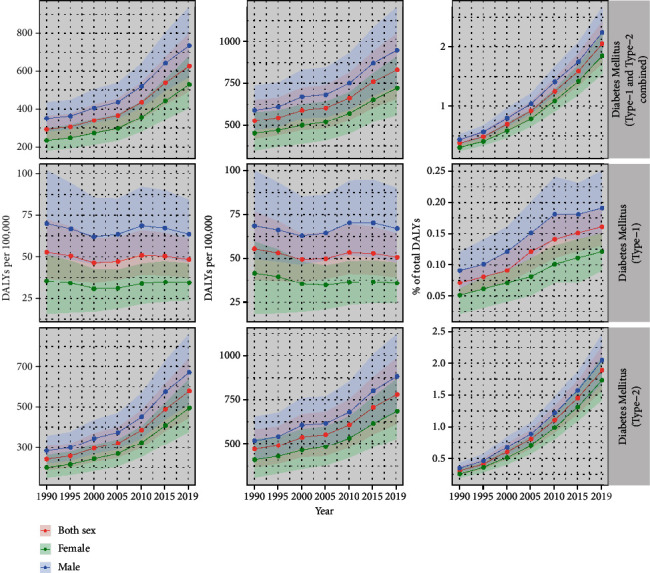
DALYs per 100,000 attributable to DM.

Like prevalence and mortality, the DALYs per 100,000 population attributable to DM have increased from 1990 to 2019. In 2019, 348 (95% UI: 277.98-434.51) DALYs per 100,000 population were attributable to DM, which is approximately 2.04% (95% UI: 1.71-2.42) of total DALYs in 2019 (Figure 1). Approximately 2.23% (95% UI: 1.85-2.66) of total DALYs in male and 1.84% (95% UI: 1.53-2.22) of total DALYs in female were due to DM in 2019. (Note: details in Table 1 of the Supplemental file Tables).

### 3.4. Age and Sex Disaggregated Prevalence, Mortality, and DALYs

**Figure 2:**
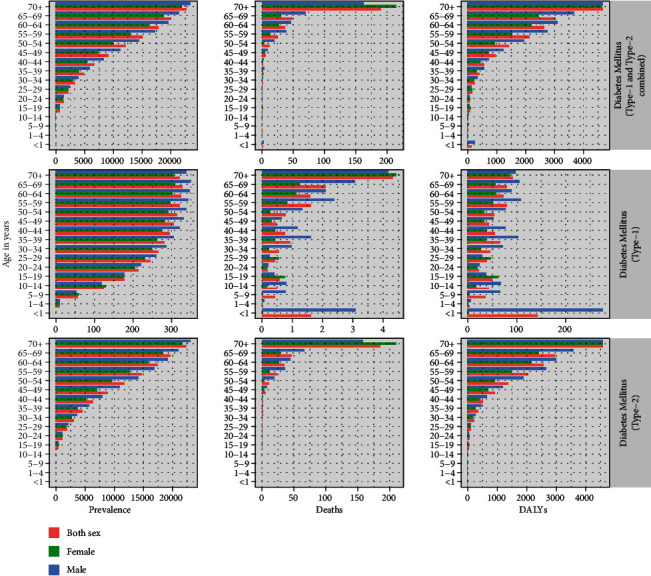
Age and sex distribution of the prevalance, mortality, and DALYs of DM.

Figure 2 shows that the burden of diabetes among participants less than 1 year of age, 1-4 years, 5-9 years, and 10-14 years is exclusively attributed by DM type 1. The prevalence, mortality, and DALY due to DM type 2 seem to increase gradually with age. The prevalence of DM type 2 increases from 584.81 per 100,000 populations in age 15-19 years to 22345.74 among participants of age seventy years and above. Similarly, deaths due to DM type 2 increase from 0.35 per 100,000 in age 15-19 years to 186.01 per 100,000 among participants of age 70 years and above, while the DALY attributable to DM type 2 increases from 56.02 per 100,000 among participants of 15-19 years to 4568.74 per 100,000 among participants of age 70 years and above. (Note: details in Table 2 of the Supplemental file Tables).

## 4. Discussion

Overall, the age-standardized prevalence of DM in Nepal increased from 3,192.87 cases per 100,000 population in 1990 to 5,735.58 cases per 100,000 population in 2019 (3.19% in 1990 and 5.73% in 2019), an increment of 79.64%. The rate is higher than that at the global (47.81%) and South Asia levels (65.94%) [22]. Globally, DM type 2 accounts for 90% of DM prevalence [7]. Similar to the global composition, the prevalence of DM type 2 is much higher than DM type 1 in Nepal. There were an estimated 1,350,744 cases of DM type 2 and 61,437 cases of DM type 1 in Nepal, with about 95.6% of the total cases being DM type 2 [22]. Hence, the increasing age-standardized prevalence of DM in Nepal is the result of an increase in DM type 2, while the prevalence of DM type 1 has remained relatively stable between 1990 and 2019. In one of the previous systematic reviews, the prevalence of type 2 DM was found to range from a minimum of 1.4% to 19.0% in different studies with a pooled prevalence of 8.4% [23]. In the other review published in 2020, the prevalence of prediabetes and DM was 9.2% and 8.5%, respectively [5]. The prevalence of DM in Nepal in our study is comparable to the previously published studies.

The risk factors for DM type 2 include obesity, overweight, family history of DM, age, history of gestational DM, and physical inactivity [24, 25]. Although obesity alone is a significant risk factor when it coalesces with physical inactivity, the risk of DM type 2 is magnified with both these risk factors combined [25–27]. The estimates from a 2019 national survey of NCD risk factors state that close to a quarter of the Nepalese adult population (24.3%) aged 15-69 years are overweight and obese (body mass index (BMI) ≥ 25 kg/m2). Further, overall burden of DM attributable to high BMI and low physical activity has also gone up between 1990 and 2019 [22].

According to the International Diabetes Federation (IDF), the largest increases in prevalence have been observed in countries which have been transiting from low- to middle-income status [7]. In the last two decades, Nepal's gross national income per capita has increased from US$ 220 (in 2001) to US$ 1,090 (in 2020), marking its move toward a middle-income nation [1]. The economic growth in countries headed for middle-income status has bolstered the prospects of reducing the burden of infectious diseases, including malnutrition [28]. However, it has unfortunately led to a rise in the burden of NCDs such as DM. The globalized economy has allowed multinational beverage and food companies to expand their products into such markets through marketing campaigns and offer choices for an array of highly processed, highly palatable, snack based, low cost, energy dense, sugar or salt rich, and obesity-promoting products that drive up obesity rates which might have some contribution to increasing DM rates [29, 30]. The expansion of the transnational food and beverage companies has also been incentivized by market saturation in developed economies and their drive to attain growth targets as a profit-making entity [29, 31, 32].

Globally, the shift from traditional plant-based diets to diets rich in unhealthy saturated fats, trans fats, sugar, salt, and increased consumption of meat and ultraprocessed food, referred to as “nutrition transition”, has been one of the factors responsible for the rise in obesity [29, 33–35]. This shift has been observed in Nepal too. It is considered the fourth stage of the transition which is marked by increasing consumption of sugar and fat-rich diet, fast food, and decreasing carbohydrate consumption [36]. This could be potentially linked to the rising burden of DM in Nepal as well. Furthermore, between 1990 and 2019, the overall burden (DALYs) of DM was attributable to dietary risks such as diets high in processed meat, a diet low in fruits, a diet low in whole grains, a diet high in red meat, diets high in sugar-sweetened beverages and has dramatically increased in Nepal [22].

Urbanization is significantly and positively associated with obesity [37]. The urban environment exposes individuals to processed foods and sugary drinks and mass media campaigns encouraging people's preference toward them, passive modes of travel, limited outdoor space for recreational activities, and more often desk jobs that facilitate a rise in obesity rates [38, 39]. Rapid increase in the urban population in Nepal [40, 41] leading to sedentary lifestyles and unhealthy foods, which could be one reason for the increasing prevalence of DM in Nepal.

### 4.1. DM Prevention

Behavior change is central to DM prevention [42]. According to the IDF, dietary modification and physical activity should be considered the primary prevention approaches [27]. Evidence suggests that moderate to vigorous physical activity reduces the risk of DM and reduces mortality among people with DM [43]. At least 150 minutes (about 2 and a half hours) per week of aerobic exercise (rhythmic movements of large muscles generally, for at least 10 minutes at a time like swimming, jogging, walking, bicycling, etc.) and at least two sessions of resistance exercise per week (brief, repetitive exercises using weight machines, resistance bands, and push-ups that could increase muscle strength) could reduce the risk of DM [44]. One of the previous studies reveals that approximately 7.4% of Nepalese population have insufficient levels of physical activity. On average, Nepalese spend 210 minutes (about 3 and a half hours) per day of physical activity [16]. Different motivation strategies like setting up specific physical activity goals, facilitating or promoting self-monitoring tools like pedometers or accelerometers, and individualized support for improving glycemic control, achieving, and sustaining weight loss can be effective [44].

Despite the importance of physical activity in reducing the risk of DM, the lack of outdoor space to perform such physical activities in cities presents a significant challenge. An earlier study suggests a positive association between levels of physical activity and the availability of green space in urban settings [45]. Hence, considering the rapid growth of the urban population in Nepal [40], it is essential to ensure that green spaces in townships of Nepal are protected as they turn into major urban centers. Although data on the level of physical activity among Nepalese population are available [2, 16], there is dearth of literature on appropriate strategies to promote physical activity among those who have insufficient physical activity level, which could be potential area for further research.

Sugar-sweetened beverages are often considered the major contributor to obesity and DM [46]. Sugar-sweetened soft drinks could provide a high amount of rapidly absorbed carbohydrates, thereby increasing the risk of DM type 2 [47]. In the nurses' health study (prospective cohort study), individuals consuming at least one sugar-sweetened beverage per day were found to have an 83% higher risk of DM compared to those consuming fewer than one beverage per month [47]. Taxation could be one option to disincentivize people from consuming sugar-sweetened beverages. In a study, a penny-per-ounce tax on sugar-sweetened beverages was found to reduce the consumption of such products by approximately 15%. The study also estimated that the resulting modest decline in BMI from taxation on sugar-sweetened beverages could result in an approximately 1.5% reduction in obesity and a 2.6% reduction in new DM cases [46]. Another study in India estimated that a 20% sugar-sweetened beverage tax could reduce obesity prevalence by 3% and DM prevalence by 1.6% between 2014 and 2023 [48]. A similar study in South Africa estimated that a 20% sugar-sweetened beverage tax could reduce the prevalence of DM type 2 by 4% over 20 years, while the associated health care cost reduction was estimated to be approximately US$ 860 million [49]. Taxation on sugary products could also be an option to generate additional resources for the health system, which can be used to care for patients with DM or expand community outreach for DM screening through peer educators or FCHVs. Results from the randomized trail demonstrated that drastic action such as preventing purchase of sugar-sweetened beverages, candies and baked items coupled with monetary incentives to purchase healthy food items such as fruits and vegetables improved nutrition quality of study participant's diet [50]. However, on financial incentive's ability to reduce purchase of unhealthy food items, the evidence is weak [51].

Further, other demand side interventions that have been used include traffic lights. It is a labelling scheme that simplifies traditional nutritional labelling methods where unhealthy food items are marked as red and healthy ones with green [52, 53]. The simplification of food labels provides an opportunity for people with low levels of literacy and numeracy skills to understand nutritional quality of food items at the point of purchase [54]. Further, around the world other labelling techniques are in use, for instance, daily intake guides health star rating, high sugar symbol labels, and health warnings. However, a recently published systematic review reports that there is inconclusive evidence to determine the effectiveness of nutrition labels in the packaging to prevent consumers from choosing healthier food items [55]. DM is primarily prevalent among adults [7], but prevention strategies should also focus on promoting healthy behaviors in children. Unhealthy food habits that can originate during childhood which is precursor to DM. For companies producing ultraprocessed food and sugar-sweetened beverages, children serve the interest as a life-long consumer of the product with whom the companies can aim to develop brand loyalty [56]. Children exhibit an innate natural liking for sweet and salty high-calorie food, [57] which helps market their products and contributes to the formation of an “obesogenic environment” [58]. The consumption behavior of children is influenced by exposure to food advertisements [59–61]. Obesogenic food products are often marketed through television, Facebook pages, mobile applications, brand websites, advergames, flash animations, music, viral marketing, [62] sponsorships, magazines, food packaging, and celebrity endorsements [61–63]. Some other marketing approaches such as sponsorship of junior sporting clubs and games fostered brand loyalty to the food product as children associate their favorite sports with food sponsors [63, 64]. The country should also have in place strategies to regulate the advertisement of unhealthy food products specially targeted at children.

### 4.2. Early Diagnosis and Treatment

However, the more significant concern is the proportion of people who are unaware of their DM status. Globally, one in two people (50.1%) living with DM are unaware that they have the disease and out of this, 84.3% of them come from low- and middle-income countries such as Nepal [1]. Previous studies reveal that seven out of every 10 adults with diabetes in Nepal are not aware of their raised blood sugar levels [7, 16]. Those people come to know about it later, leading to serious complications, disability, and even premature death. A study in 2019 also reveals that among those who were aware of their diabetic status, 5.9% had been treated, while other 14.7% who were under treatment did not have blood pressure under control which could be concerning figures for policy makers and program implementers [16]. In 2019, a total of 3,245 deaths were estimated to have occurred from DM type 2, and 288 deaths were estimated to have occurred from DM type 1 [22]. Furthermore, in resource constraint settings such as Nepal, the diabetes care and treatment is challenged by service-side barriers such as lack of human resources for screening activities especially at the community level, absence of Nepal specific guidelines for disease management, lack of required drugs in health facilities, unavailability of laboratory equipment required for routine test [65]. Similarly, the demand-side barriers such as geographical inaccessibility, the need to travel long distances to get a health facility providing specialized care, and the financial burden of managing the disease make DM management even more challenging [65, 66]. Studies on cost of care on DM in Nepal report that in the range of US$ 18.74 to US$ 65.60 per month [65, 67]. Likewise, disease management and treatment are often jeopardized by a lack of awareness about comorbidities associated with the disease and even limitations in available treatment [68]. Furthermore, people with the disease may lack financial resources to purchase essential medications and undertake dietary modifications required for disease management, [66, 69] potentially responsible for aggravating complications and even death.

Care of diabetic patients could be expensive considering hospitalizations, outpatient care, and medications that could increase disease prevalence. DM could pose a substantial economic burden to the patient, family, and the country. However, the impact of DM in terms of associated health complications and costs could be reduced by preventing the disease through lifestyle and dietary modifications and early diagnosis of the disease [70]. Strategies that are intended to prevent and control DM could also be effective in reducing the burden of other NCDs like cardiovascular diseases, cancer, etc., as many NCDs share common risk factors.

### 4.3. DM Control

Some innovative models can be explored to reduce the burden of DM in Nepal. The peer educator model was found effective in controlling DM at the community level. Peer educators served as an intermediary between physicians and hard-to-reach patients and were assigned the roles of arranging village-level health meetings to discuss lifestyle and dietary modifications, reaching out to patients, explaining the blood results, and monitoring the blood sugar level for a minimum payment of US$ 0.04 per kilometer for travel [71]. Nepal may use the existing network of female community health volunteers (FCHVs) in reaching the community for lifestyle and dietary modification discussions at the community level after basic training on the topic. One of the previous studies has revealed that the vast network of female community health volunteers (FCHVs) could be an option for DM management in a rural setting if trained properly [72].

A nurse can also keep DM under control, serving as a liaison between patient and physician by educating patients and ensuring adherence through follow up. In resource-limited settings, nurses may also assume a physician's responsibilities in managing DM, which can be facilitated by appropriate training and detailed management protocols. They may also serve to encourage patients to make lifestyle modifications, intake of healthy diets, quit smoking, and adopting regular physical exercise, which could further help achieve good glycemic control and reduce the chances of developing complications [73].

One of the previous studies revealed that the patient's financial constraints and the lack of access to health services and medication could hinder achieving reasonable diabetic control [74]. Expanding service and population coverage of the current national health insurance scheme could reduce financial barriers to access to services. This could also be an opportunity to generate additional resources in the health system, which could be further used to ensure access through expansion of services and ensuring a continuous supply of medicines.

### 4.4. DM in the Health Care Delivery System in Nepal

With the introduction of the new constitution of Nepal in 2015, the country has entered a unitary system with three spheres of government structure: federal, provincial, and local level government (LLG) [63]. Similarly, the country has developed policies for delivering all Nepali citizens with universal access to health care and providing every citizen with the constitutional right to free basic health services from the state, emergency health services, and equal access to health services [75]. The health care system is managed by various levels of health service delivery units and governance structures across the three spheres of government. The LLGs are the ones responsible for delivering “basic” health services. The overall health program in Nepal is guided by the National Health Policy 2019 and the Nepal Health Sector Strategy 2015-2022 which are in line with the major priorities of sustainable development goals putting universal health coverage at its centers [75–77]. In addition to the National Health Policy and the Health Sector Strategy prioritizing NCDs for prevention and control, which also includes DM, the National Multisectoral Action Plan for the Prevention and Control of NCDs (2014–2020) highlights cardiovascular diseases, DM, chronic respiratory diseases, and cancer among other NCDs that need attention and aims to have 25% relative reduction in overall mortality from cardiovascular diseases, cancers, DM, or chronic respiratory diseases by 2025 [78]. Further to this, to strengthen the health system, particularly the primary care system, the Nepalese Ministry of Health and Population has launched the package of essential noncommunicable diseases (PEN) program in the country initially through pilot implementation and a gradual scale up later. The PEN program targets DM as the priority condition for early detection and management at the peripheral health care level [79]. This program has adopted the PEN protocol and has a detailed protocol specifying both dietary and lifestyle interventions and medicinal management of DM, especially for the primary care level [79]. In the essential free drug list, metformin is included to be available in peripheral health facilities [80].

### 4.5. Limitations of the Study

One of the main limitations of the BOD estimates is the availability of age, sex, and year disaggregated primary data. In case data are not available, the results are based on out-of-sample predictive validity statistical modelling. As the data for the GBD analysis were pulled from multiple sources, the rigor of the original study and measurement process could influence the results [19].

### 4.6. Scope for Further Research

More precise estimation on the risk factors of DM type 2 could help policy makers to tailor the intervention based on country context. For example, some of the previous studies suggest that air pollution can increase the risk of DM type 2, [81, 82] which has largely been ignored in rolling out interventions for prevention of DM type 2. Additional data, particularly on environmental risk factors like indoor and outdoor air pollution (including data on PM 2.5 with larger geographical coverage) and the effect of high and low temperatures on human health, may be helpful to further improve the estimations on NBoD. As the risk of unfavorable outcomes increases with increase in multimorbidity, further studies on prevalence, risk factors of cooccurrence of diabetes with other chronic diseases could be useful.

## 5. Conclusions

There has been considerable increase in prevalence, mortality rate, and DALYs attributable to DM in Nepal which could further increase in the future posing a serious challenge to the health system. Health systems need to prepare themselves to deal with the higher number of DM cases that require chronic long-term care. Prevention of DM requires collaborative efforts from multiple sectors for risk factors controlling interventions such as promotion of physical activity and consumption of a healthy diet. At the same time, the health sector needs to be responsive toward accelerating the early diagnosis and treatment of DM. The current federal structure could be an opportunity for integrated, locally tailored public health and clinical interventions for the prevention of the disease and its consequences.

## Figures and Tables

**Table 1:** Prevalence of DM (per 100,000 population) from 1990 to 2019.

Sex	Year	DM		DM type 1		DM type 2	
		All-age prevalence rate (95% UI)	Age-standardized prevalence rate (95% UI)	All-age prevalence rate (95% UI)	Age-standardized prevalence rate (95% UI)	All-age prevalence rate (95% UI)	Age-standardized prevalence rate (95% UI)
Both sexes	1990	1,861.58 (1,678.6-2,058.88)	3,192.87 (2,883.68-3,516.35)	154.53 (118.22-197.56)	187.79 (146.09-237.53)	1,707.06 (1,520.34-1,918.68)	3,005.07 (2,697.35-3,332.37)
	1995	2,055.27 (1,873.6-2,247.97)	3,465.61 (3,168.3-3,774.27)	155.57 (119.68-197.92)	187.82 (146.84-236.3)	1,899.69 (1,713.04-2,102.42)	3,277.79 (2,984.09-3,591)
	2000	2,511.54 (2,285.67-2,754.5)	4,098.03 (3,744.94-4,478.75)	160.06 (122.44-204.37)	189.45 (147.15-239.61)	2,351.48 (2,119.97-2,597.95)	3,908.58 (3,551.29-4,292.01)
	2005	2,684.95 (2,479.56-2,905.02)	4,160.16 (3,860.1-4,486.63)	166.35 (127.58-210.43)	191.28 (148.23-240.42)	2,518.6 (2,316.39-2,734.15)	3,968.88 (3,654.32-4,289.59)
	2010	3,109.54 (2,873.67-3,361.6)	4,441.38 (4,116.19-4,778.95)	176.79 (135.56-224.79)	196.5 (152.25-247.4)	2,932.76 (2,698.8-3,190.14)	4,244.88 (3,920.96-4,582.18)
	2015	3,812.16 (3,510.86-4,135.7)	5,015.67 (4,634.53-5,435.54)	189.32 (145.16-239.11)	203.37 (157.15-257.07)	3,622.84 (3,319.8-3,956)	4,812.3 (4,433.09-5,237.36)
	2019	4,642.83 (4,178.58-5,137.74)	5,735.58 (5,168.74-6,327.73)	201.99 (156.94-255.48)	211.49 (165.51-265.12)	4,440.84 (3,983.96-4,937.08)	5,524.09 (4,969.32-6,119.99)

Male	1990	2,057.96 (1,843.02-2,278.83)	3,460.66 (3,118.874-3,806.04)	159.5 (122.7-202.39)	196.49 (153.06-246.85)	1,898.46 (1,677.49-2,123.72)	3,264.17 (2,910.2-3,624.93)
	1995	2,274.11 (2,047.24-2,511.52)	3,786.889 (3,424.5-4,148.55)	160.49 (124.28-206.04)	196.47 (153.91-248.28)	2,113.63 (1,888.77-2,343.43)	3,590.42 (3,227.95-3,960.92)
	2000	2,843.96 (2,570.03-3,151.28)	4,592.31 (4,160.368-5,072.57)	165.61 (126.79-212.89)	198.57 (154.45-250.06)	2,678.35 (2,400.98-2,974.78)	4,393.73 (3,958.85-4,878.64)
	2005	3,058.69 (2,825.52-3,315.65)	4,672.487 (4,317.361-5,041.52)	170.49 (130.47-218.05)	199.25 (154.27-249.43)	2,888.2 (2,646.46-3,133.89)	4,473.24 (4,115.53-4,828.07)
	2010	3,513.7 (3,228.94-3,811.89)	4,966.136 (4,574.607-5,357.26)	181.72 (138.82-231.42)	206.21 (159.71-258.91)	3,331.98 (3,044.2-3,628.99)	4,759.93 (4,364.42-5,165.62)
	2015	4,295.94 (3,933.59-4,682.69)	5,663.751 (5,192.54-6,162.29)	194.24 (149.2-247.47)	214.14 (167.32-271.82)	4,101.7 (3,738.71-4,498.35)	5,449.62 (4,983.82-5,956.78)
	2019	5,133.27 (4,609-5,694.48)	6,440.782 (5,762.38-7,144.47)	207.42 (161.33-264.1)	223.55 (174.91-279.97)	4,925.85 (4,396.85-5,521.46)	6,217.23 (5,556.46-6,923.94)

Female	1990	1,664.21 (1,493.95-1,856.64)	2,912.25 (2,610.97-3,216.85)	149.53 (113.28-192.7)	179.11 (137.79-228.47)	1,514.68 (1,335.85-1,718.71)	2,733.14 (2,431.11-3,053.46)
	1995	1835.641 (1,650.78-2,032.55)	3133.01 (2,833.74-3,442.86)	150.64 (114-193.32)	179.22 (138.03-226.38)	1685 (1,501.37-1,881.95)	2953.8 (2,651.54-3,263.94)
	2000	2,178.25 (1,967.80-2,418.66)	3,589.15 (3,266.77-3,953.19)	154.49 (119.19-198.64)	180.37 (140.71-228.26)	2,023.76 (1,805.01-2,271.2)	3,408.78 (3,076.76-3,783.66)
	2005	2,318.091 (2,102.76-2529.83)	3,643 (3,324.57-3,966.88)	162.28 (124.23-206.93)	183.56 (141.98-230.46)	2,155.81 (1,958.99-2,368.62)	3,459.44 (3,158.36-3,781.56)
	2010	2,725.14 (2,495.157-2,980.709)	3936.21 (3,623.35-4,270.87)	172.09 (130.49-219.32)	187.6 (143.9-235.52)	2,553.05 (2,326.15-2,801.17)	3,748.61 (3,427.47-4,085.31)
	2015	3,364.31 (3,063.43-3,708.70)	4,427.04 (4,040.81-4,872.36)	184.77 (141.21-233.04)	194.07 (149.63-243.18)	3,179.54 (2,886.6-3,507.98)	4,232.97 (3,842.42-4,662.56)
	2019	4,196.157 (3,734.80-4,678.26)	5,125.94 (4,573.8-5,707.68)	197.04 (152.65-252.94)	201.46 (156.7-256.25)	3,999.12 (3,539.54-4,487.43)	4,924.48 (4,374.84-5,506.53)

**Table 2:** Mortality rate from DM from 1990 to 2019.

Sex	Year	DM			DM type 1			DM type 2		
		All-age mortality rate (95% UI)	Age-standardized mortality rate (95% UI)	% of total deaths in both sex (95% UI)	All-age mortality rate (95% UI)	Age-standardized mortality rate (95% UI)	% of total deaths in both sex (95% UI)	All-age mortality rate (95% UI)	Age-standardized mortality rate (95% UI)	% of total deaths in both sex (95% UI)
Both sex	1990	4.85 (3.93-5.99)	12.05 (9.63-14.75)	0.43 (0.36-0.5)	0.74 (0.44-1.07)	0.96 (0.51-1.45)	0.07 (0.04-0.09)	4.11 (3.27-5.14)	11.09 (8.83-13.74)	0.36 (0.3-0.44)
	1995	4.98 (4.17-6.04)	12.2 (10.1-14.69)	0.54 (0.47-0.62)	0.71 (0.44-0.99)	0.91 (0.51-1.32)	0.08 (0.05-0.1)	4.27 (3.55-5.23)	11.29 (9.33-13.63)	0.46 (0.39-0.55)
	2000	5.21 (4.46-6.08)	12.35 (10.57-14.41)	0.7 (0.62-0.81)	0.64 (0.43-0.88)	0.84 (0.5-1.18)	0.09 (0.06-0.12)	4.56 (3.87-5.45)	11.51 (9.78-13.5)	0.61 (0.53-0.73)
	2005	5.95 (5.03-6.96)	13.12 (11.03-15.28)	0.92 (0.79-1.06)	0.66 (0.47-0.9)	0.84 (0.55-1.17)	0.1 (0.07-0.14)	5.29 (4.44-6.28)	12.28 (10.37-14.35)	0.82 (0.7-0.96)
	2010	7.61 (6.48-8.95)	15.15 (12.89-17.68)	1.22 (1.07-1.41)	0.74 (0.53-1.02)	0.92 (0.63-1.27)	0.12 (0.09-0.16)	6.87 (5.82-8.17)	14.23 (12.07-16.69)	1.1 (0.96-1.3)
	2015	10.06 (8.29-12.11)	18.41 (15.12-21.89)	1.52 (1.32-1.77)	0.77 (0.54-1.06)	0.94 (0.66-1.31)	0.12 (0.09-0.16)	9.29 (7.62-11.22)	17.47 (14.35-20.84)	1.41 (1.21-1.65)
	2019	11.42 (9.02-13.88)	19.57 (15.5-23.58)	1.8 (1.54-2.07)	0.75 (0.54-1.03)	0.91 (0.64-1.26)	0.12 (0.09-0.16)	10.67 (8.43-12.99)	18.66 (14.66-22.57)	1.68 (1.44-1.95)

Male	1990	5.67 (4.29-7.28)	11.91 (9-15.19)	0.48 (0.38-0.58)	0.98 (0.66-1.52)	1.15 (0.71-1.91)	0.08 (0.06-0.13)	4.69 (3.5-6.1)	10.77 (8.07-13.78)	0.39 (0.31-0.49)
	1995	5.71 (4.6-6.99)	11.9 (9.59-14.62)	0.58 (0.49-0.69)	0.93 (0.64-1.4)	1.09 (0.7-1.74)	0.1 (0.06-0.14)	4.77 (3.8-6.04)	10.81 (8.71-13.54)	0.49 (0.4-0.6)
	2000	5.87 (4.88-7.05)	11.97 (9.93-14.53)	0.73 (0.63-0.86)	0.87 (0.61-1.25)	1.03 (0.68-1.57)	0.11 (0.08-0.15)	5.01 (4.08-6.13)	10.94 (8.95-13.42)	0.62 (0.52-0.75)
	2005	6.5 (5.22-7.86)	12.29 (9.85-14.96)	0.91 (0.76-1.09)	0.9 (0.63-1.28)	1.05 (0.69-1.59)	0.13 (0.09-0.18)	5.6 (4.43-6.91)	11.25 (8.86-13.77)	0.79 (0.63-0.96)
	2010	8.33 (6.76-10.2)	14.27 (11.6-17.42)	1.19 (0.99-1.42)	1.01 (0.7-1.47)	1.17 (0.76-1.75)	0.14 (0.1-0.2)	7.32 (5.92-9.03)	13.1 (10.67-16.21)	1.05 (0.85-1.26)
	2015	11.02 (8.4-13.94)	17.38 (13.32-21.83)	1.45 (1.16-1.74)	1.04 (0.67-1.5)	1.21 (0.73-1.78)	0.14 (0.09-0.19)	9.98 (7.58-12.7)	16.17 (12.34-20.42)	1.31 (1.04-1.6)
	2019	12.39 (9.08-15.88)	18.38 (13.55-23.35)	1.7 (1.35-2.05)	1.01 (0.63-1.49)	1.16 (0.7-1.81)	0.14 (0.09-0.2)	11.38 (8.28-14.68)	17.22 (12.66-22.03)	1.56 (1.23-1.91)

Female	1990	4.02 (2.95-5.35)	11.5 (8.21-15.22)	0.37 (0.28-0.48)	0.5 (0.12-0.78)	0.75 (0.15-1.18)	0.05 (0.01-0.07)	3.52 (2.52-4.9)	10.75 (7.51-14.34)	0.05 (0.01-0.07)
	1995	4.25 (3.31-5.45)	11.98 (9.08-15.46)	0.49 (0.39-0.61)	0.48 (0.14-0.72)	0.71 (0.18-1.09)	0.05 (0.02-0.08)	3.77 (2.9-4.99)	11.27 (8.58-14.71)	0.05 (0.02-0.08)
	2000	4.53 (3.71-5.58)	12.2 (9.78-15.14)	0.66 (0.55-0.8)	0.42 (0.15-0.61)	0.62 (0.19-0.92)	0.06 (0.02-0.09)	4.11 (3.35-5.15)	11.57 (9.3-14.35)	0.06 (0.02-0.09)
	2005	5.42 (4.5-6.56)	13.35 (10.75-16.53)	0.93 (0.79-1.12)	0.43 (0.18-0.61)	0.61 (0.23-0.9)	0.07 (0.03-0.1)	4.99 (4.1-6.13)	12.74 (10.2-15.77)	0.07 (0.03-0.1)
	2010	6.93 (5.75-8.4)	15.39 (12.51-18.69)	1.26 (1.06-1.49)	0.48 (0.24-0.7)	0.66 (0.31-0.95)	0.09 (0.04-0.12)	6.45 (5.39-7.79)	14.74 (12.02-17.94)	0.09 (0.04-0.12)
	2015	9.17 (7.39-11.2)	18.8 (14.63-23.39)	1.61 (1.32-1.94)	0.52 (0.27-0.75)	0.69 (0.35-1.01)	0.09 (0.05-0.13)	8.65 (6.94-10.65)	18.11 (14.13-22.45)	0.09 (0.05-0.13)
	2019	10.54 (8.15-13.25)	20.16 (15.03-25.53)	1.91 (1.56-2.34)	0.52 (0.28-0.75)	0.68 (0.35-0.99)	0.09 (0.05-0.13)	10.02 (7.76-12.68)	19.48 (14.5-24.63)	0.09 (0.05-0.13)

Note: all-age and age-standardized mortality rate is expressed as deaths per 100,000 population.

## Data Availability

The exact data presented in the results section can be accessed from https://ghdx.healthdata.org/gbd-results-tool?params=gbd-api-2019-permalink/05478a3260aab7cab558ab383eb2279a. The link above has been generated from GBD results tool (https://ghdx.healthdata.org/gbd-results-tool) page in the IHME's website. In the link, we selected the location Nepal. Year:1990, 1995, 2000, 2005, 2010, 2015, and 2019; Context: cause; Age: all ages and age standardized; Metrics: percent and rate; Measures: deaths, DALYs, and prevalence; Sex: male, female, and both; Cause: diabetes mellitus, diabetes mellitus type I, and diabetes mellitus type II.

## Supplementary Materials

Supplementary Materials This paper has one supplementary file. (1) Tables: it consists of two tables which consist of detailed data from which Figures 1 and 2 in the paper have been populated. The table titles are as follows. (a) Table 1: DALYs per 100,000 population attributable to DM. (b) Table 2: age and sex distribution of the prevalence, mortality, and DALY of the diabetes mellitus.
