# Supplementary material for: Burden of Diabetes Mellitus in Nepal: An Analysis of Global Burden of Disease Study 2019
Source: J Diabetes Res. 2022 Dec 20;2022:4701796. doi: 10.1155/2022/4701796 (PMC9794432; doi:10.1155/2022/4701796)
Supplement: Supplementary Materials — This paper has one supplementary file. (1) Tables: it consists of two tables which consist of detailed data from which Figures 1 and 2 in the paper have been populated. The table titles are as follows. (a) Table 1: DALYs per 100,000 population attributable to DM. (b) Table 2: age and sex distribution of the prevalence, mortality, and DALY of the diabetes mellitus. [file 4701796.f1.docx]

**Table 1:** DALYs per 100,000 population attributable to DM

|  |  | **DM** | | | **DM type 1** | | | **DM type 2** | | |
| --- | --- | --- | --- | --- | --- | --- | --- | --- | --- | --- |
| **Sex** | **Year** | **All-****age DALYs**  **per 100,000**  **(95% UI)** | **Age-standardized**  **DALY per 100,000**  **(95% UI)** | **% Of total**  **DALY (95% UI)** | **All-****age DALYs**  **per 100,000**  **(95% UI)** | **Age-standardized**  **DALY per 100,000**  **(95% UI)** | **% Of total**  **DALY (95% UI)** | **All-=ages DALY**  **per 100,000**  **(95% UI)** | **Age-standardized**  **DALY per 100,000**  **(95% UI)** | **% Of total**  **DALY (95% UI)** |
| **Both sex** | 1990 | 290  (231.56-361.34) | 520.79  (413.21-647.15) | 0.37  (0.3-0.44) | 52.34  (36.31-71.88) | 55.24  (37.29-76.3) | 0.07  (0.05-0.09) | 237.66  (183.35-302.47) | 465.54  (364.91-579.16) | 0.3  (0.24- 0.36) |
|  | 1995 | 303.17  (243.3-373.54) | 539.24  (435.61-661.35) | 0.48  (0.4-0.58) | 50.11  (35.31-67.91) | 52.99  (36.75-70.86) | 0.08  (0.06-0.1) | 253.06  (196.38-315.89) | 486.25  (383.69-596.97) | 0.4  (0.32- 0.49) |
|  | 2000 | 336.9  (268.73-422.48) | 584.41  (465.67-720.55) | 0.69  (0.57-0.83) | 46.09  (33.93-60.88) | 49.1  (36.13-63.82) | 0.09  (0.07-0.12) | 290.81  (224.68-367.74) | 535.31  (420.55-667.02) | 0.6  (0.48- 0.73) |
|  | 2005 | 364.5  (287.72-453.98) | 599.36  (475.24-735.55) | 0.91  (0.76-1.07) | 46.84  (35.63-61.32) | 49.47  (37.9-64.32) | 0.12  (0.09-0.15) | 317.66  (243.58-399.21) | 549.89  (427.59-679.71) | 0.8  (0.65- 0.95) |
|  | 2010 | 433.71  (344.95-538.23) | 658.53  (525.51-811.93) | 1.24  (1.05-1.46) | 50.49  (38.72-65.58) | 53.01  (40.81-69.24) | 0.14  (0.11-0.18) | 383.22  (295.14-482.35) | 605.51  (474.94-751.19) | 1.1  (0.9- 1.32) |
|  | 2015 | 536.16  (421.46-672.04) | 756.24  (599.54-938.73) | 1.58  (1.33-1.88) | 50.02  (38.39-64.11) | 52.75  (40.26-67.79) | 0.15  (0.12-0.18) | 486.14  (377.66-613.93) | 703.49  (551.5-875.76) | 1.44  (1.19- 1.72) |
|  | 2019 | 623.76  (489.83-796.74) | 826.94  (654.37-1045.86) | 2.04  (1.71-2.42) | 47.91  (37.06-60.63) | 50.55  (38.82-64.08) | 0.16  (0.13-0.19) | 575.86  (443-737.27) | 776.39  (608.71-980.86) | 1.88  (1.57- 2.26) |
| **Male** | 1990 | 348  (277.98-434.51) | 584.58  (466.13-733.79) | 0.43  (0.35-0.52) | 69.54  (50.35-101.46) | 68.33  (49.5-99.67) | 0.09  (0.06-0.12) | 278.46  (215.38-354.41) | 516.25  (402.74-652.23) | 0.34  (0.27- 0.42) |
|  | 1995 | 360.53  (289.13-446.42) | 605.55  (483.01-750.04) | 0.56  (0.46-0.67) | 66.38  (48.01-94.19) | 65.98  (47.97-92.87) | 0.1  (0.08-0.14) | 294.15  (229.89-374.18) | 539.57  (425.51-674.55) | 0.45  (0.37- 0.56) |
|  | 2000 | 403.46  (323.83-502.64) | 666.25  (536.01-827.62) | 0.79  (0.65-0.96) | 61.62  (45.27-85.13) | 62.53  (46.28-84.88) | 0.12  (0.09-0.16) | 341.84  (263.36-434.31) | 603.72  (470.57-757.42) | 0.67  (0.54- 0.84) |
|  | 2005 | 434.84  (347.26-536.39) | 678.19  (542.26-841.66) | 1.03  (0.86-1.21) | 63.14  (47.53-84.59) | 64.04  (48.39-85.91) | 0.15  (0.11-0.2) | 371.7  (285.71-468.2) | 614.15  (476.31-765.12) | 0.88  (0.71- 1.06) |
|  | 2010 | 517.12  (405.82-639.9) | 747.3  (587.59-927.32) | 1.4  (1.17-1.66) | 68.3  (51.45-91.71) | 69.93  (51.44-93.95) | 0.18  (0.14-0.24) | 448.83  (342.65-567.77) | 677.37  (526.49-850.51) | 1.21  (0.99- 1.47) |
|  | 2015 | 639.19  (492.8-801.87) | 866.84  (671.92-1079.84) | 1.74  (1.44-2.07) | 66.89  (49.29-89.51) | 69.94  (50.51-94.42) | 0.18  (0.14-0.23) | 572.31  (436.5-726.81) | 796.9  (611.05-1,003.34) | 1.56  (1.28- 1.87) |
|  | 2019 | 730.8  (564.77-934.54) | 944.73  (728.51-1203.16) | 2.23  (1.85-2.66) | 63.21  (46.39-84.01) | 66.8  (47.48-89.66) | 0.19  (0.15-0.25) | 667.59  (508.87-858.12) | 877.93  (674.84-1117.5) | 2.04  (1.66- 2.45) |
| **Female** | 1990 | 231.7  (178.76-295.6) | 447.14  (343.54-572.55) | 0.3  (0.24-0.37) | 35.05  (15.17-51.06) | 41.21  (17.87-59.34) | 0.05  (0.02-0.06) | 196.64  (146.82-257.99) | 405.93  (306.17-524.91) | 0.25  (0.19- 0.33) |
|  | 1995 | 245.61  (191.13-313.26) | 465.22  (367.29-584.85) | 0.4  (0.33-0.49) | 33.79  (16.01-47.17) | 39.23  (18.31-55.22) | 0.06  (0.03-0.08) | 211.82  (160.48-273.96) | 425.99  (330.12-540.37) | 0.35  (0.27- 0.43) |
|  | 2000 | 270.16  (209.56-344.15) | 494.74  (384.73-622.02) | 0.58  (0.46-0.72) | 30.52  (16.61-42.4) | 35.01  (19.18-47.89) | 0.07  (0.04-0.09) | 239.64  (181.01-310.55) | 459.74  (355.25-582.43) | 0.51  (0.4- 0.64) |
|  | 2005 | 295.45  (230.75-370.67) | 513.84  (404.94-638.33) | 0.78  (0.65-0.93) | 30.83  (18.53-41.96) | 34.34  (20.47-45.67) | 0.08  (0.05-0.11) | 264.62  (201.88-336.61) | 479.5  (373.91-596.72) | 0.7  (0.57- 0.85) |
|  | 2010 | 354.38  (278.59-439.94) | 566.27  (445.12-693.29) | 1.08  (0.9-1.28) | 33.55  (21.33-45.65) | 35.98  (23.16-48.26) | 0.1  (0.07-0.13) | 320.83  (248.74-402.46) | 530.29  (417.59-654.2) | 0.98  (0.8- 1.19) |
|  | 2015 | 440.77  (342.23-557.97) | 648.31  (513.97-807.7) | 1.41  (1.18-1.69) | 34.4  (22.61-46.91) | 36.18  (24.22-48.75) | 0.11  (0.07-0.14) | 406.37  (311.82-517.87) | 612.13  (475.48-763.64) | 1.3  (1.08- 1.58) |
|  | 2019 | 526.28  (403.04-676.02) | 717.85  (555.83-907.97) | 1.84  (1.53-2.22) | 33.97  (23.22-45.73) | 35.38  (24.23-47.46) | 0.12  (0.09-0.15) | 492.31  (373.55-636.09) | 682.47  (527.12-865.05) | 1.72  (1.41- 2.09) |

**Table 2:** Age and sex distribution of the prevalence, mortality, and DALY of the diabetes mellitus

| Sex | Age in  years | Prevalence (95% UI) | | | Deaths (95% UI) | | | DALY (95% UI) | | |
| --- | --- | --- | --- | --- | --- | --- | --- | --- | --- | --- |
|  |  | DM | DM 1 | DM 2 | DM | DM 1 | DM 2 | DM | DM 1 | DM 2 |
| Both sex | <1 | 0  (0, 0) | 0  (0, 0) | 0  (0, 0) | 1.62  (0.65, 3.44) | 1.62  (0.65, 3.44) |  | 143.13  (57.48, 304.27) | 143.13  (57.48, 304.27) | 0  (0, 0) |
|  | 1-4 | 11.51  (5.52, 19.22) | 11.51  (5.52, 19.22) | 0  (0, 0) | 0.04  (0.02, 0.13) | 0.04  (0.02, 0.13) |  | 4.41  (1.86, 12) | 4.41  (1.86, 12) | 0  (0, 0) |
|  | 5-9 | 57.9  (30.64, 93.79) | 57.9  (30.64, 93.79) | 0  (0, 0) | 0.42  (0.22, 0.72) | 0.42  (0.22, 0.72) |  | 36.8  (20.45, 60.5) | 36.8(20.45, 60.5) | 0  (0, 0) |
|  | 10-14 | 124.8  (77.4, 183.22) | 124.8  (77.4, 183.22) | 0  (0, 0) | 0.49  (0.26, 0.84) | 0.49  (0.26, 0.84) |  | 43.9  (26.23, 71.59) | 43.9  (26.23, 71.59) | 0  (0, 0) |
|  | 15-19 | 762.81  (572.04, 999.83) | 178  (123.38, 245.37) | 584.81  (374.15, 826.48) | 0.95  (0.51, 1.47) | 0.58  (0.3, 0.94) | 0.37  (0.18, 0.64) | 106.72  (69.91, 150.06) | 50.7  (30.28, 76.82) | 56.02  (36.17, 84.09) |
|  | 20-24 | 1390.78  (1100.92, 1765.04) | 213.47  (158.54, 284.33) | 1177.31  (876.69, 1549.18) | 0.27  (0.17, 0.41) | 0.18  (0.1, 0.28) | 0.09  (0.05, 0.16) | 91.93  (61.55, 133.33) | 23.2  (15.94, 32.45) | 68.73  (42.53, 106.33) |
|  | 25-29 | 2274.76  (1824.61, 2830.98) | 244.66  (187.34, 318.2) | 2030.1  (1579.15, 2579.18) | 0.72  (0.43, 1.15) | 0.43  (0.24, 0.71) | 0.3  (0.16, 0.53) | 169.48  (117.87, 231.45) | 39.63  (26.09, 57.99) | 129.85  (83.98, 187.83) |
|  | 30-34 | 3450.57  (2846.5, 4179.42) | 265.45  (203.21, 340.72) | 3185.13  (2590.06, 3914.83) | 1.23  (0.59, 2) | 0.56  (0.25, 0.97) | 0.68  (0.31, 1.16) | 273.22  (191.59, 376.9) | 47.18  (28.32, 73.63) | 226.04  (155.72, 320.86) |
|  | 35-39 | 4929.25  (4119.94, 5801.8) | 281.47  (215.67, 358.59) | 4647.78  (3847.28, 5543.26) | 2.19  (0.99, 3.46) | 0.93  (0.38, 1.58) | 1.26  (0.57, 2.1) | 434.07  (303.97, 589.82) | 66.47  (35.48, 100.95) | 367.6  (251.4, 514.69) |
|  | 40-44 | 6781.97  (5718.42, 7965.07) | 295.09  (228.52, 374.71) | 6486.88  (5415.41, 7676.61) | 2.08  (1.23, 3.45) | 0.76  (0.41, 1.31) | 1.32  (0.74, 2.41) | 580.36  (399.67, 808.11) | 56.6  (37.87, 83.98) | 523.76  (346.75, 738.43) |
|  | 45-49 | 9223.33  (7922.27, 10642.63) | 305.96  (238.78, 384.75) | 8917.37  (7622.6, 10348.18) | 6.44  (3.99, 9.56) | 0.47  (0.24, 0.86) | 5.97  (3.68, 8.88) | 982.47  (693.13, 1346.79) | 43.36  (28.64, 65.93) | 939.11  (662.23, 1293.97) |
|  | 50-54 | 12143.09  (10422.41, 14055.65) | 314.04  (245.62, 395.97) | 11829.05  (10117.4, 13729.82) | 11.89  (7.63, 19.2) | 0.78  (0.36, 1.53) | 11.1  (7.12, 18.06) | 1443.77  (1038.59, 1983.29) | 55.14  (35.29, 85.93) | 1388.64  (995.03, 1911.14) |
|  | 55-59 | 15077.41  (13147.21, 17284.93) | 320.27  (254.47, 401.12) | 14757.14  (12862.14, 16975.77) | 25.54  (16.28, 38.19) | 1.61  (0.79, 2.92) | 23.94  (15.25, 35.89) | 2140.88  (1595.55, 2842.13) | 80.17  (50.28, 124.26) | 2060.71  (1536.87, 2749.43) |
|  | 60-64 | 17810.19  (15588.56, 20280.31) | 324.93  (260.89, 403.58) | 17485.26  (15286.14, 19961.55) | 36.4  (23.98, 53.1) | 1.59  (0.79, 2.76) | 34.81  (22.87, 50.47) | 2643.14  (1950.6, 3491.41) | 74.16  (49.32, 110.1) | 2568.98  (1888.72, 3405.64) |
|  | 65-69 | 19993.58  (17581.29, 22570.02) | 327.74  (263.02, 404.92) | 19665.84  (17275.98, 22203.72) | 48.56  (32.03, 69.96) | 2.11  (1.12, 3.68) | 46.46  (30.4, 66.87) | 3043.15  (2221.38, 4022.48) | 80.52  (51.78, 118.49) | 2962.64  (2161.55, 3923.48) |
|  | 70+ | 22667.03  (20200.86, 25182.59) | 321.29  (259.12, 393.98) | 22345.74  (19906.03, 24901.89) | 190.33  (148.24, 232.22) | 4.32  (2.49, 6.59) | 186.01  (144.77, 226.24) | 4661.37  (3741.28, 5773.03) | 92.63  (63.57, 128.92) | 4568.74  (3661.74, 5648.7) |
| Male | <1 | 0  (0, 0) | 0  (0, 0) | 0  (0, 0) | 3.11  (1.21, 6.62) | 3.11  (1.21, 6.62) |  | 275.75  (107.4, 585.57) | 275.75  (107.4, 585.57) | 0  (0, 0) |
|  | 1-4 | 10.9  (5.36, 18.41) | 10.9  (5.36, 18.41) | 0  (0, 0) | 0.07  (0.02, 0.24) | 0.07  (0.02, 0.24) |  | 6.89  (2.24, 21.49) | 6.89  (2.24, 21.49) | 0  (0, 0) |
|  | 5-9 | 54.11  (28.56, 87.22) | 54.11  (28.56, 87.22) | 0  (0, 0) | 0.79  (0.41, 1.38) | 0.79  (0.41, 1.38) |  | 66.88  (35.88, 113.99) | 66.88  (35.88, 113.99) | 0  (0, 0) |
|  | 10-14 | 119.78  (71.22, 176.53) | 119.78  (71.22, 176.53) | 0  (0, 0) | 0.81  (0.4, 1.44) | 0.81  (0.4, 1.44) |  | 68.13  (36.3, 116.2) | 68.13  (36.3, 116.2) | 0  (0, 0) |
|  | 15-19 | 718.75  (529.56, 954.17) | 179.23  (123.36, 247.39) | 539.52  (334.83, 785.18) | 0.56  (0.32, 0.89) | 0.41  (0.22, 0.68) | 0.15  (0.07, 0.28) | 76.75  (52.98, 108.6) | 38.53  (24.24, 58.84) | 38.22  (23.14, 58.57) |
|  | 20-24 | 1406.29  (1094.19, 1815.45) | 221.98  (165.85, 295.87) | 1184.31  (862.04, 1585.99) | 0.26  (0.12, 0.46) | 0.2  (0.09, 0.36) | 0.06  (0.02, 0.12) | 91.5  (58.69, 135.13) | 24.89  (15.47, 37.2) | 66.62  (38.5, 104.14) |
|  | 25-29 | 2483.1  (1961.33, 3184.91) | 260.53  (197.59, 339.75) | 2222.57  (1690.54, 2912.71) | 0.34  (0.16, 0.59) | 0.25  (0.12, 0.44) | 0.09  (0.04, 0.18) | 156.06  (101.37, 227.29) | 29.68  (18.56, 44.34) | 126.38  (76.62, 193.26) |
|  | 30-34 | 4041.8  (3260.48, 4971.05) | 286.69  (219.06, 370.87) | 3755.11  (2975.91, 4685.59) | 2.14  (0.79, 3.72) | 0.98  (0.34, 1.91) | 1.16  (0.39, 2.14) | 358.05  (240.78, 498.45) | 72.54  (33.89, 126.74) | 285.5  (192.06, 406.24) |
|  | 35-39 | 6012.13  (5015.13, 7217.22) | 305.93  (233.69, 393.88) | 5706.2  (4683.67, 6912.41) | 3.6  (1.2, 6.19) | 1.61  (0.49, 3.04) | 1.98  (0.64, 3.7) | 578.67  (385.27, 807.83) | 103.41  (42.82, 178.09) | 475.26  (311.6, 665.08) |
|  | 40-44 | 8383.38  (7026.14, 9986.73) | 320.79  (244.66, 410.24) | 8062.59  (6691.05, 9676.71) | 3.02  (1.58, 5.04) | 1.17  (0.48, 2.2) | 1.85  (0.84, 3.28) | 745.02  (501.26, 1045.86) | 77.75  (43.23, 128.86) | 667.27  (432, 949.91) |
|  | 45-49 | 11283.18  (9668.5, 13098.24) | 331.72  (255.47, 419.45) | 10951.46  (9364.42, 12774.6) | 8.82  (4.87, 14.12) | 0.64  (0.22, 1.42) | 8.18  (4.51, 13.2) | 1256.89  (886.86, 1760.74) | 53.09  (31.4, 89.81) | 1203.8  (843.91, 1674.14) |
|  | 50-54 | 14502.93  (12552.17, 16593.42) | 339.19  (264.56, 427.59) | 14163.74  (12192.36, 16260.42) | 20.19  (12.24, 31.66) | 1.33  (0.5, 2.83) | 18.86  (11.2, 29.43) | 1968.09  (1417.63, 2644.88) | 78.09  (44.36, 135.59) | 1890.01  (1356.82, 2552) |
|  | 55-59 | 17301.74  (15064.32, 19775.98) | 344.64  (272.87, 433.44) | 16957.1  (14754.87, 19444.36) | 38.49  (22.59, 62.08) | 2.42  (0.9, 5.1) | 36.07  (21.05, 58.4) | 2776.22  (2021.55, 3765.88) | 109.19  (57.58, 195.2) | 2667.03  (1927.42, 3602.07) |
|  | 60-64 | 19557.74  (17204.36, 22054.2) | 348.34  (278.18, 435.71) | 19209.4  (16859.47, 21762.36) | 46.09  (27.43, 71.71) | 2.09  (0.83, 4.34) | 43.99  (26.38, 68.6) | 3098.2  (2239.9, 4145.69) | 90.71  (52.53, 155.78) | 3007.49  (2177.54, 4032.16) |
|  | 65-69 | 21398.82  (18865.98, 24058.86) | 349.83  (279.82, 435.03) | 21048.99  (18519.35, 23615.09) | 69.28  (41.93, 103.36) | 3.09  (1.24, 6.12) | 66.19  (39.89, 99.26) | 3688.56  (2627.92, 4887.18) | 105.82  (57.45, 179.89) | 3582.74  (2559.57, 4742.26) |
|  | 70+ | 23461.11  (20857.95, 26097.45) | 338.29  (271.96, 417.5) | 23122.83  (20515.05, 25744.13) | 163.16  (116.16, 214.46) | 4.17  (1.92, 7.95) | 158.99  (113.58, 208.07) | 4664.72  (3570.16, 5893.05) | 98.28  (59.56, 164.03) | 4566.44  (3498.29, 5747.51) |
| Female | <1 | 0  (0, 0) | 0  (0, 0) | 0  (0, 0) | 0.03  (0.01, 0.08) | 0.03  (0.01, 0.08) |  | 2.76  (1.05, 6.75) | 2.76  (1.05, 6.75) | 0  (0, 0) |
|  | 1-4 | 12.15  (5.83, 20.86) | 12.15  (5.83, 20.86) | 0  (0, 0) | 0.01  (0.01, 0.02) | 0.01  (0.01, 0.02) |  | 1.8  (0.96, 3.02) | 1.8  (0.96, 3.02) | 0  (0, 0) |
|  | 5-9 | 61.89  (32.69, 101.1) | 61.89  (32.69, 101.1) | 0  (0, 0) | 0.03  (0.01, 0.06) | 0.03  (0.01, 0.06) |  | 5.21  (2.5, 8.82) | 5.21  (2.5, 8.82) | 0  (0, 0) |
|  | 10-14 | 129.98  (82.93, 188.76) | 129.98  (82.93, 188.76) | 0  (0, 0) | 0.16  (0.03, 0.35) | 0.16  (0.03, 0.35) |  | 18.86  (6.74, 33.68) | 18.86  (6.74, 33.68) | 0  (0, 0) |
|  | 15-19 | 805.69  (606.83, 1048.55) | 176.8  (123.91, 246.68) | 628.89  (419.68, 879.22) | 1.33  (0.37, 2.27) | 0.75  (0.17, 1.38) | 0.58  (0.22, 1.08) | 135.89  (70.92, 209.23) | 62.54  (21.5, 107.22) | 73.35  (43.2, 114.43) |
|  | 20-24 | 1377.21  (1089.3, 1732.25) | 206.03  (151.36, 274.18) | 1171.18  (876.32, 1532.5) | 0.28  (0.17, 0.46) | 0.16  (0.08, 0.29) | 0.12  (0.06, 0.23) | 92.29  (61.34, 137) | 21.72  (13.52, 31.98) | 70.57  (44.58, 110.26) |
|  | 25-29 | 2109.63  (1706.03, 2606.2) | 232.08  (174.46, 300.78) | 1877.56  (1479.01, 2376.32) | 1.02  (0.55, 1.77) | 0.56  (0.26, 1.05) | 0.46  (0.22, 0.89) | 180.12  (126.07, 247.54) | 47.52  (27.2, 78.58) | 132.6  (87.22, 188.92) |
|  | 30-34 | 3007.78  (2499.13, 3629.53) | 249.53  (190.42, 321.75) | 2758.25  (2245.23, 3371.8) | 0.56  (0.29, 0.93) | 0.24  (0.1, 0.44) | 0.32  (0.15, 0.6) | 209.68  (145.13, 294.21) | 28.18  (17.71, 42.08) | 181.51  (121.51, 261.88) |
|  | 35-39 | 4115.28  (3428.76, 4835.31) | 263.09  (200, 338.26) | 3852.19  (3163.08, 4588.96) | 1.13  (0.63, 1.77) | 0.42  (0.18, 0.76) | 0.71  (0.37, 1.23) | 325.37  (225.36, 454.05) | 38.7  (24.1, 59.9) | 286.67  (194.46, 410.43) |
|  | 40-44 | 5505.97  (4603.65, 6478.81) | 274.61  (211.3, 349.56) | 5231.37  (4328.36, 6188.37) | 1.34  (0.75, 3.09) | 0.44  (0.19, 0.84) | 0.9  (0.47, 2.26) | 449.15  (301.95, 651.75) | 39.74  (24.78, 60.7) | 409.41  (267.53, 598.83) |
|  | 45-49 | 7445.16  (6242.18, 8695.84) | 283.72  (220.96, 359.01) | 7161.44  (5947.42, 8402.5) | 4.39  (2.58, 7.24) | 0.32  (0.12, 0.64) | 4.07  (2.37, 6.83) | 745.57  (506.59, 1044.46) | 34.97  (20.72, 53.07) | 710.61  (481.49, 991.1) |
|  | 50-54 | 9956.61  (8371.81, 11819.24) | 290.74  (228.07, 365.95) | 9665.87  (8065.77, 11547.63) | 4.19  (2.35, 11.68) | 0.27  (0.1, 0.6) | 3.92  (2.2, 10.85) | 957.97  (651.43, 1456.04) | 33.87  (20.52, 54.4) | 924.09  (619.33, 1397.74) |
|  | 55-59 | 12972.12  (11083, 15226.51) | 297.21  (234.56, 372.33) | 12674.91  (10792.31, 14908.32) | 13.29  (7.87, 22.34) | 0.83  (0.31, 1.66) | 12.45  (7.4, 21.02) | 1539.55  (1098.37, 2111.76) | 52.7  (30.68, 84.17) | 1486.84  (1056.63, 2031.5) |
|  | 60-64 | 16196.32  (13890.46, 18744.93) | 303.31  (240.59, 376.98) | 15893  (13582.59, 18417.61) | 27.45  (16.03, 44.47) | 1.13  (0.41, 2.2) | 26.32  (15.45, 43.1) | 2222.88  (1596.75, 3066.82) | 58.88  (34.7, 92.19) | 2164.01  (1558.68, 2981.6) |
|  | 65-69 | 18749.46  (16349.83, 21674.22) | 308.18  (245.29, 380.64) | 18441.28  (15992.31, 21362.02) | 30.22  (18.05, 59.17) | 1.24  (0.45, 2.42) | 28.98  (17.22, 57.1) | 2471.75  (1750.99, 3483.16) | 58.12  (35.48, 91.24) | 2413.63  (1710.57, 3387.72) |
|  | 70+ | 21930.67  (19346.86, 24666.28) | 305.53  (246.76, 373.8) | 21625.13  (19055.28, 24373.99) | 215.53  (157.52, 279.92) | 4.45  (1.93, 7) | 211.08  (154.11, 273.32) | 4658.27  (3660.51, 5860.77) | 87.4  (51.66, 123.28) | 4570.87  (3578.66, 5766.88) |
